# Detecting Associations between Early-Life DDT Exposures and Childhood Growth Patterns: A Novel Statistical Approach

**DOI:** 10.1371/journal.pone.0131443

**Published:** 2015-06-30

**Authors:** Brianna Heggeseth, Kim Harley, Marcella Warner, Nicholas Jewell, Brenda Eskenazi

**Affiliations:** 1 Department of Mathematics and Statistics, Williams College, Williamstown, MA, United States of America; 2 Center for Environmental Research and Children’s Health, School of Public Health, University of California, Berkeley, California, United States of America; 3 Division of Biostatistics, School of Public Health, University of California, Berkeley, California, United States of America; University of Rennes-1, FRANCE

## Abstract

It has been hypothesized that environmental exposures at key development periods such as in utero play a role in childhood growth and obesity. To investigate whether in utero exposure to endocrine-disrupting chemicals, dichlorodiphenyltrichloroethane (DDT) and its metabolite, dichlorodiphenyldichloroethane (DDE), is associated with childhood physical growth, we took a novel statistical approach to analyze data from the CHAMACOS cohort study. To model heterogeneity in the growth patterns, we used a finite mixture model in combination with a data transformation to characterize body mass index (BMI) with four groups and estimated the association between exposure and group membership. In boys, higher maternal concentrations of DDT and DDE during pregnancy are associated with a BMI growth pattern that is stable until about age five followed by increased growth through age nine. In contrast, higher maternal DDT exposure during pregnancy is associated with a flat, relatively stable growth pattern in girls. This study suggests that in utero exposure to DDT and DDE may be associated with childhood BMI growth patterns, not just BMI level, and both the magnitude of exposure and sex may impact the relationship.

## Introduction

The number of obese individuals has drastically increased recently worldwide [1]. Obesity is likely caused by a complex combination of genetic, behavioral, and environmental factors. While much attention has been placed on curtailing overeating and encouraging physical activity, there have also been many attempts to understand the biological mechanism behind the disease. Researchers have attempted to quantify the role of heredity using twin studies [2] and more recently, it has been suggested that early environmental factors may play a role. In particular, prenatal exposure to endocrine-disrupting compounds (EDCs) is hypothesized to deregulate the metabolic system, disrupt growth regulation, and increase the risk of childhood obesity [3, 4]. One EDC is the pesticide dichlorodiphenyltrichloroethane (DDT) and its metabolite, dichlorodiphenyldichloroethane (DDE).

Animal toxicological studies support the potential role of DDT and DDE on growth of organisms [5–7]; however, results of studies in humans have been inconsistent [8] with some studies suggesting no associations with body mass index (BMI) [9–11] and others suggesting a positive relationship [12–14]. In addition, there is evidence to suggest that the relationship between DDT and DDE and growth may be non-linear [12] and sex-specific due to differences in endocrine activity [12, 15–18].

Most research on the relationship between early life exposure and physical development has focused on BMI measured at a single time point, such as at ages 14 months [13], 14–22 months [19], 6.5 years [12], 7 years [20, 21] and 9 years [18]. This approach inherently focuses only on the BMI level and assumes that there is an age at which the impact of chemical exposure on growth becomes evident ignoring any potential impact on the growth over time. Other studies examine repeated measures of BMI over time, modeling the relationship between prenatal chemical exposure and BMI using standard longitudinal approaches such as linear mixed effects models and marginal models [9, 14, 20]. While the focus is still typically on the relationships with the magnitude of BMI levels, researchers explore relationships with growth by modeling the linear model coefficients using an interaction term or with secondary regression [22]. This multi-level structure restricts the relationship between exposure and growth pattern to be modeled through slope coefficients, which does not easily accommodate complex, non-linear relationships. Standard practice involves including one interaction term between exposure and age to allow the coefficient of age to depend linearly on exposure levels. For example, a positive coefficient for an interaction between exposure and age would indicate that increased exposure is associated with a higher rate of change assuming all other variables to be constant. This drastically limits the flexibility of the model.

In contrast, a more data-driven approach, using a finite mixture model, uses subgroups to model the heterogeneity in the growth patterns [23]. Risk factors such as chemical exposure can be used to model group membership probabilities. This framework does not restrict the form of the relationship between exposure and developmental groups; it has the flexibility to model non-linear growth patterns as well as non-linear relationships between the groups and the exposure level. These methods are becoming more commonly used in practice due to their flexibility and the availability of software such as Proc Traj [24], Mplus [25], and R packages such as lcmm [26].

This mixture model involves constructing subgroups according to the growth patterns to enhance the study of associations between exposures and the change over time. Numerous studies have recently used this data-driven approach to study early life factors associated with physical growth [27–31]. However, with a continuous outcome such as BMI, these mixture model-based methods iteratively define subgroups by minimizing the variability around estimated group trajectory means. Therefore, they primarily group individuals by the *level* of BMI rather than on the *shape* of their developmental BMI pattern over time. Thus, these studies may have missed correctly estimating and detecting interesting relationships with growth patterns. A solution that we propose is to first transform the outcome by subtracting individual-specific means prior to using existing mixture model methods in order to temporarily remove the level in order to focus on growth [32]. We have previously shown through simulation that this approach performs better than the standard mixture models and other clustering methods in detecting an appropriate number of developmental pattern groups with small misclassification error. With little misclassification in group membership, estimating the relationships between exposures and growth pattern groups is more accurate. The study of associations with exposures on the overall level can complement this proposed analysis of the shapes of growth patterns.

In this study, we present a novel statistical analysis of the association of a well-known endocrine disruptor, the pesticide DDT and its metabolite DDE, with trajectories of childhood BMI in the Center for the Health Assessment of Mothers and Children of Salinas (CHAMACOS) study, a longitudinal birth cohort study in an agricultural community in California. Additionally, we illustrate the effectiveness of a transformed mixture modeling approach to study the relationship of maternal prenatal exposure with child BMI growth patterns from age 2 to 9 years.

## Methods

### Study participants

The CHAMACOS Study is a longitudinal birth cohort study investigating the health effects of environmental chemicals on pregnant women and their children living in an agricultural region of California. Pregnant women were recruited to participate in 1999 and 2000 from six prenatal clinics serving the farmworker population of the Salinas Valley, California. Eligible women were less than 20 weeks gestation at enrollment, at least 18 years of age, qualified for low-income health insurance (Medicaid), spoke English or Spanish, and were planning to deliver at the county hospital. A total of 527 women were enrolled and followed through the birth of a live-born, singleton infant. Serum concentrations of DDT and DDE during pregnancy were collected for 415 women. Of those mother-child pairs, complete anthropometric data were collected on 306 children at age 2, 270 at age 3.5, 264 at age 5, 268 at age 7, and 260 at age 9. We included the 250 children with complete anthropometric data from 4 of the 5 study visits between ages 2 and 9. We excluded one child due to a health condition known to lead to weight loss. This group of 249 children made up the analytic sample for this study. Written informed consent was obtained from all women for their participation and from parents or guardians on behalf of the children to participate. Additionally, verbal assent was recorded from the children starting at age 7. All informed consent and study protocols were approved by University of California Berkeley’s Committee for the Protection of Human Subjects 1 (University of California Berkeley IRB #1: Registration #: IRB00000455).

### Procedure

Details of the study have been previously published [33]. Mothers were interviewed twice during pregnancy (at approximately 13 and 26 weeks gestation), at delivery, and when their children were 0.5, 1, 2, 3.5, 5, 7, and 9 years of age. Mothers were asked about their sociodemographic and health characteristics, including education, family income, country of birth, years of residence in the United States, pre-pregnancy weight, and smoking status. Information was also gathered on child dietary and health habits, including duration of breastfeeding, consumption of soda, sugary snacks, and fast food, and exercise and sedentary behavior.

Birth weight was abstracted from medical records. Children were measured at every visit. Weight, rounded to the nearest 0.1 kg, was measured using a digital scale between ages 2 and 7 years (Tanita Mother-Baby scale, model 1582; Tanita Corp., Arlington Heights, IL) and a foot-to-foot bioimpedance scale starting at age 9 years (Tanita TBF-300A Body Composition Analyzer, Tanita Corporation of America, Inc., Arlington Heights, Illinois). Height was measured to the nearest 0.1 cm using a stadiometer. All measurements were made in triplicate and averaged for analysis.

### Laboratory analysis

Concentrations of *o,p*’-DDT, *p,p*’-DDT, and *p,p*’-DDE were measured in maternal serum collected at approximately 26 weeks gestation (n = 230) or delivery (n = 19). Serum samples were stored at -80 C° until shipment to the Centers for Disease Control and Prevention (CDC) for analysis using isotope dilution gas chromatography-high resolution mass spectrometry [34]. Quality control samples were included in each run. Concentrations below the limit of detection (LOD) were assigned the value one-half the LOD [35]. Maternal serum was analyzed for total cholesterol and triglyceride levels using standard enzymatic methods (Roche Chemicals, Indianapolis, IN). Measured DDT and DDE values were lipid-adjusted and reported as ng/g of lipid [36].

### Statistical analysis

We calculated BMI as weight (kilograms) divided by height (meters) squared from the assessments conducted when the children were approximately 2, 3.5, 5, 7, and 9 years old. We used BMI values rather than BMI z-scores from sex- and age-specific BMI charts published by the CDC since the charts are derived from cross-sectional data and may not accurately represent typical BMI growth patterns [37]. Lipid-adjusted levels of *o,p*’-DDT, *p,p*’-DDT, and *p,p*’-DDE were log10-transformed and analyzed as continuous variables. Descriptive statistics were calculated; median and interquartile range (IQR) were used for variables with non-symmetric distributions.

Rather than trying to model BMI development using time-varying explanatory variables, we used a finite number of groups to account for the heterogeneity in BMI growth and then studied the relationship between baseline risk factors and these developmental groups. To directly focus on the growth pattern, or trajectory shape, we transformed the BMI measurements by subtracting individual-specific means and then we fit a finite multivariate Gaussian mixture model with the transformed BMI repeated measures as the outcome measurements (*y*). Conditional on the child’s age (*t*) and baseline risk factors (*z*), the probability density function for this mixture model is a weighted sum of densities representing K subgroups, *f*(*y*|*t*, *z*) = *π*_1_(*z*)*f*_1_(*y*|*t*) + ⋯ + *π*_*K*_(*z*)*f*_*K*_(*y*|*t*), where *π*_1_(*z*), …, *π*_*K*_(*z*) are group membership probabilities modeled using a multinomial logistic regression with baseline risk factors as predictors and *f*_1_, …, *f*_*k*_ are multivariate Gaussian densities with distinct (unknown) means and covariance matrices to account for longitudinal dependencies. The baseline risk factors of interest, the log10-transformed maternal serum concentrations of *o,p*’-DDT, *p,p*’-DDT, and *p,p*’-DDE, were included in a multinomial logic function along with other possible confounding demographic variables to model group probabilities, *π*_*k*_(*z*). The group mean growth patterns over time were modeled using a quadratic B-spline [38] with one internal knot at the median.

Initially, the model was estimated without any baseline risk factors. Model parameters and posterior group probabilities for models with *K* = 2, 3, 4, and 5 groups were estimated using the expectation maximization (EM) algorithm [39] assuming independence and then the exponential correlation structure, both assuming constant variance. The optimal number of groups, *K*, and the correlation structure were selected based on the data such that the Bayesian Information Criterion (BIC) was minimized. Individual children were classified into the group with the largest estimated posterior group probability for visual representation.

Once the number of groups, K, and the covariance structure were chosen, baseline risk factors were included as predictors for the group membership probabilities and the overall model was re-estimated. For each group *j* = 1, …, *K*−1, relative risk ratios (RRR),

$$RRR=\frac{P(\text{Group}\;j|z+1)/P(\text{Group}\;j|z)}{P(\text{Group}\;K|z+1)/P(\text{Group}\;K|z)}$$ (1)

were calculated for one-unit (ten-fold) increases in maternal *o,p*’-DDT, *p,p*’-DDT, and *p,p*’-DDE serum concentrations during pregnancy based on estimated parameters and presented with 95% confidence intervals (CI) based on asymptotic normality.

We stratified by sex and adjusted for possible confounding baseline factors in the multinomial regression predicting the group membership probabilities. The factors, including the number of years in the United States, self-reported maternal pre-pregnancy BMI, child’s birth weight, and duration of breastfeeding, were previously identified as possible predictors of exposure and BMI [18, 20]. Interactions between these baseline variables and the main risk factor, DDT or DDE exposure, were considered individually to see if the relationship between the risk factor depended on the child or maternal characteristics. Sensitivity analyses were completed with regard to preterm birth (gestational age < 37 months) and low birth weight (birth weight < 2500 g). All statistical analyses were performed using R [40], version 3.0.2.

## Results

### Population

Of the mother-child pairs with serum concentrations, the children who had at least four BMI measurements between ages 2 and 7 (n = 249) did not differ in sex distribution, BMI at baseline age 2, maternal DDT or DDE exposure, gestational age, the number of years in the USA, and duration of breastfeeding from those who did not (n = 165). The mothers missing maternal DDT or DDE concentrations and therefore excluded from this analysis had been living in the US significantly longer, breastfed a shorter period of time, and had children with slightly lower birth weights on average than those in the sample.

Table 1 describes the analytical sample in terms of baseline maternal and child characteristics. Most of the mothers were born in Mexico (91%), had not finished a high school education (80%), were married or living as married (85%), and had lived in the US at least two years prior to enrolling in the study (76%, median: 5.1 years, IQR: 10-1.75 years). Before pregnancy, the mean BMI was 27.7 kg/m^2^ (SD: 5.6 kg/m^2^) and 65% of the mothers were overweight or obese, and after delivery, almost all of the mothers breastfed for at least 2 weeks (93%), continuing for a median of 7 months (IQR: 13-3 months).

**Table 1:** Baseline Child and Maternal Characteristics of Study Population (n = 249), Center for the Health Assessment of Mothers and Children of Salina Study, 2000–2010.

**Characteristic**		**No. (%)**
Child sex	Male	113 (45.4)
	Female	136 (54.6)
Country of maternal birth	USA	23 (9.2)
	Mexico/Other	226 (90.8)
Years of maternal residence in USA	Median, IQR	5.1, 10-1.75
	≤ 5	129 (51.8)
	> 5	120 (48.2)
Maternal education	≤ 6th grade	109 (43.8)
	7th–12th grade	89 (35.7)
	> High school	51 (20.5)
Maternal marital status	Not married	38 (15.3)
	Married/living as married	211 (84.7)
Maternal pre-pregnancy BMI (kg/m^2^)	Mean, SD	27.7, 5.6
	≤ 18.5	2 (1.0)
	18.5–24.9	85 (34.1)
	25.0–29.9	98 (39.3)
	≥ 30.0	64 (25.7)
Child birth weight (g)	Mean, SD	3.4, 0.5
	< 2500g	8 (3.2)
	2500–4200g	220 (88.4)
	> 4200g	21 (8.4)
Breastfeeding duration (months)	Median, IQR	7, 13-3
	0–1.9	38 (15.3)
	2–5.9	66 (26.5)
	6–11.9	63 (25.3)
	≥ 12	82 (32.9)

IQR, interquartile range

The children weighed an average of 3,500 g at birth and 55% were female. Table 2 describes the BMI of the 249 children in the analytical sample over time. At age 2, about 30% of the children were overweight or obese, meaning that they were at, or above, the 85th percentile on the age and sex-specific CDC growth charts [41] for BMI with mean BMI of 17.4 kg/m^2^ (SD: 2.0 kg/m^2^) at age 2. This percentage increased as the children aged to 50%, 53%, 54% and 57% at age 3.5, 5, 7, and 9 years old, respectively, with mean BMI of 20.8 kg/m^2^ (SD: 4.8 kg/m^2^) at age 9.

**Table 2:** Distribution of dhildhood BMI at 2, 3.5, 5, 7, and 9 years of age at follow-up, Center for the Health Assessment of Mothers and Children of Salina Study, 2000–2010.

	**Age at follow up (years)**				
**BMI (kg/m^2^)**	**2 (n = 240) No. (%)**	**3.5 (n = 243) No. (%)**	**5 (n = 246) No. (%)**	**7 (n = 245) No. (%)**	**9 (n = 233) No. (%)**
Mean (SD)	17.4 (2.0)	17.7 (2.7)	17.9 (3.2)	19.1 (4.0)	20.8 (4.8)
Normal^1^	167 (70)	122 (50)	116 (47)	113 (46)	101 (43)
Overweight^2^	31 (13)	46 (19)	49 (20)	45 (18)	37 (16)
Obese^3^	42 (17)	75 (31)	81 (33)	87 (36)	95 (41)

^1^ < 85th percentile

^2^ 85th–94.9th percentile

^3^ ≥ 95th percentile

BMI, body mass index; SD, standard deviation

### Exposure

Almost all of the mothers had serum concentrations of DDT and DDE during pregnancy above the limit of detection for *o,p*’-DDT (97%), *p,p*’-DDT (100%), and *p,p*’-DDE (100%). The geometric mean (geometric SD) serum levels were 1.7 (4.3) ng/g of lipid *o,p*’-DDT, 21.2 (5.3) ng/g of lipid *p,p*’-DDT, and 1,428 (3.4) ng/g of lipid *p,p*’-DDE. As reported previously [42], the observed levels were significantly higher among mothers who were born in Mexico, had lived in the United States less than 2 years, were less educated (≤ 6th grade), and who breastfed for a longer duration. There were no significant differences by maternal pre-pregnancy BMI, marital status, infant birth weight, nor sex.

### Growth patterns

**Fig 1:**
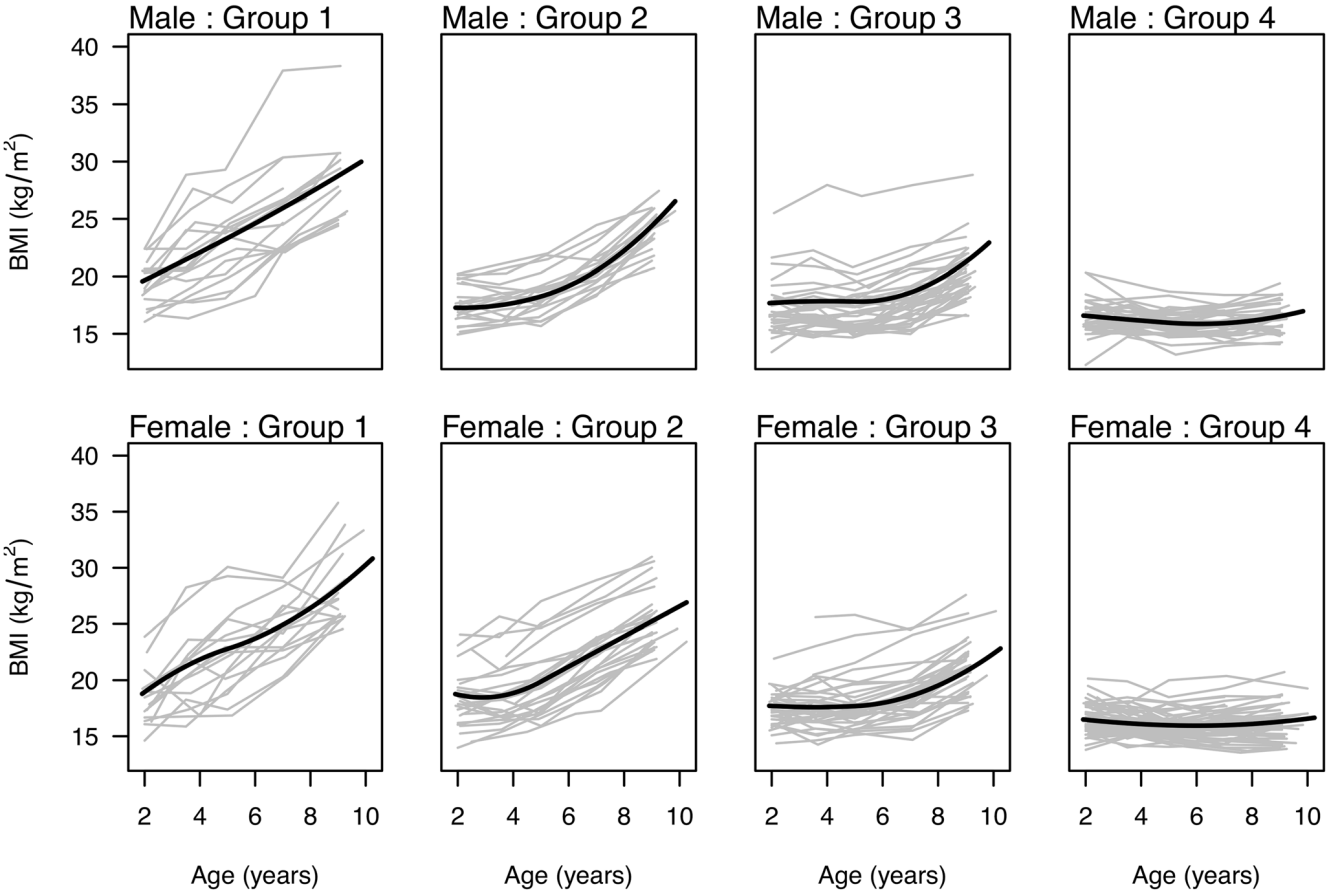
Group BMI growth patterns. BMI longitudinal trajectories of children in study population, categorized by sex and data-driven groups based on posterior probabilities from an estimated finite mixture model without adjusting for baseline risk factors. Group mean BMI trajectories are overlaid for each sex-specific group.

Four groups (*K* = 4) were chosen with independent correlation structure for both boys and girls using the BIC to select a mixture model for the transformed data. The parameter estimates were used to calculate the mean growth patterns and the posterior group probabilities were used to categorize the children into the groups according to the highest posterior probability for presentation (Fig 1). For both sexes, the four groups can be described by their mean growth pattern: group 1) linearly increasing, group 2) stable and increasing at age 4 to 5, group 3) stable and increasing at age 6 to 7, and group 4) flat and stable from age 2 until age 9.

The groups detected by the transformed mixture model and displayed in Fig 1 were well separated with little overlap in group classifications. Only about 7 to 12% of the posterior group probabilities were between 0.1 and 0.9 for each group; values close to 0 and 1 indicate more certainty in the membership while values close to 0.5 indicate a child’s pattern is in between two groups. This indicates that we can be confident that these four groups well approximate the heterogeneity in growth patterns in this data set. Based on these memberships, Table 3 shows the number of children placed in each group and describes the BMI levels across time for individuals within each growth pattern group. Group 4, which was the largest group, includes children whose patterns most resembles the 50th percentile BMI curve for American children [41] and has the highest percentage of children classified as normal weight at age 9. Conversely, the children in group 1 had quite a bit of variation in their BMIs at age 2 but were all obese by age 9, reflecting this smaller group’s pattern of steep linear increase in BMI over the study period.

**Table 3:** Sex-specific distributions (boys, girls) of childhood BMI at 2, 3.5, 5, 7, and 9 years of age at follow-up for growth pattern groups detected by transformed mixture model, Center for the Health Assessment of Mothers and Children of Salina Study, 2000–2010.

	**Age at follow up (years)**				
**BMI (kg/m^2^)**	**2 years No. (%)**	**3.5 years No. (%)**	**5 years No. (%)**	**7 years No. (%)**	**9 years No. (%)**
Group 1 (n = 18, 16)					
Mean (SD)	19 (2)^1^, 18 (2)^2^	22 (4), 21 (4)	23 (4), 23 (4)	26 (4), 25 (3)	28 (4), 28 (4)
Normal^3^	5 (27), 7 (44)	1 (5), 1 (6)	0 (0), 0 (0)	0 (0), 0 (0)	0 (0), 0 (0)
Overweight^4^	4 (22), 3 (19)	2 (11), 2 (13)	1 (6), 2 (13)	0 (0), 0 (0)	0 (0), 0 (0)
Obese^5^	9 (50), 6 (37)	14 (82), 12 (80)	17 (94), 14 (87)	18 (100), 15 (100)	13 (100), 16 (100)
Group 2 (n = 20, 26)					
Mean (SD)	18 (2), 18 (3)	18 (2), 19 (3)	19 (2), 20 (3)	22 (2), 23 (3)	24 (2), 25 (3)
Normal	13 (65), 11 (46)	7 (37), 9 (34)	74 (21), 3 (12)	0 (0), 1 (4)	0 (0), 0 (0)
Overweight	1 (5), 5 (21)	2 (11), 3 (12)	4 (21), 7 (27)	4 (17), 3 (12)	1 (5), 1 (4)
Obese	6 (30), 8 (33)	10 (53), 14 (54)	11 (58), 16 (61)	19 (83), 22 (84)	19 (95), 24 (96)
Group 3 (n = 35, 37)					
Mean (SD)	17 (2), 17 (2)	18 (3), 18 (2)	17 (2), 18 (2)	18 (3), 19 (2)	20 (2), 21 (2)
Normal	27 (77), 21 (60)	17 (50), 15 (42)	17 (49), 13 (35)	16 (47), 11 (30)	7 (20), 8 (22)
Overweight	4 (11), 9 (26)	9 (26), 10 (28)	8 (23), 15 (41)	11 (32), 17 (46)	17 (49), 16 (44)
Obese	4 (11), 5 (14)	8 (24), 11 (30)	10 (28), 9 (24)	7 (21), 9 (24)	11 (31), 12 (33)
Group 4 (n = 40, 57)					
Mean (SD)	17 (1), 16 (1)	16 (1), 16 (1)	16 (1), 16 (1)	16 (1), 16 (1)	16 (1), 16 (1)
Normal	35 (92), 48 (89)	29 (74), 43 (75)	33 (83), 46 (84)	36 (90), 49 (88)	34 (97), 52 (98)
Overweight	1 (3), 4 (7)	8 (21), 10 (18)	5 (13), 7 (13)	4 (10), 6 (11)	1 (3), 1 (2)
Obese	2 (5), 2 (4)	2 (5), 4 (7)	2 (5), 2 (4)	0 (0), 1 (2)	0 (0), 0 (0)

^1^ Boys listed first

^2^ Girls listed second

^3^ < 85th percentile

^4^ 85th–94.9th percentile

^5^ ≥ 95th percentile

BMI, body mass index; SD, standard deviation

### Association between DDT and DDE and developmental patterns

We then expanded the model to include maternal serum concentration of DDT or DDE as a baseline risk factor to predict group membership, *π*(*z*), via the multinomial logistic function. The full mixture model was refit and relative risk ratios (RRR) were estimated with and without adjusting for other possible confounding baseline factors (Table 4). By investigating interaction terms in our data, we found that baseline characteristics such as duration of breastfeeding, birth weight, maternal pre-pregnancy BMI, and number of years in the USA had no significant impact on the relationships between DDT or DDE exposure and developmental pattern group; therefore, we present the results from a model without any interaction terms.

**Table 4:** Estimated relative risk ratios (95% CI) comparing each group to the referent Group 4 for ten-fold increase in maternal serum concentrations (ng/g of lipid) of *o,p*’-DDT, *p,p*’-DDT and *p,p*’-DDE with and without adjusting for baseline risk factors.

	**Boys**			**Girls**		
	**Group 1**	**Group 2**	**Group 3**	**Group 1**	**Group 2**	**Group 3**
*o,p*’-DDT						
Unadjusted	2.4 (0.7, 8.5)	7.9 (1.7, 36.8)*	5.3 (1.2, 23.6)*	0.5 (0.1, 1.7)	0.9 (0.3, 2.7)	0.8 (0.4, 1.3)
Adjusted^1^	1.5 (0.2, 10.3)	5.1 (0.5, 55.2)	3.1 (0.3, 34.7)	0.1 (0.0, 0.7)*	0.9 (0.3, 2.9)	0.5 (0.2, 1.0)*
*p,p*’-DDT						
Unadjusted	1.7 (0.6, 5.3)	3.9 (1.2, 12.4)*	3.1 (1.0, 10.0)*	0.5 (0.2, 1.3)	0.9 (0.4, 2.1)	0.8 (0.5, 1.4)
Adjusted	1.2 (0.3, 4.5)	2.9 (0.7, 12.4)	2.1 (0.5, 8.8)	0.2 (0.1, 1.0)*	1.0 (0.4, 2.9)	0.6 (0.3, 1.2)
*p,p*’-DDE						
Unadjusted	1.2 (0.4, 3.8)	3.6 (1.1, 12.2)*	2.6 (0.8, 8.4)	0.3 (0.1, 1.4)	0.8 (0.2, 2.6)	0.8 (0.4, 1.8)
Adjusted	1.0 (0.3, 3.0)	2.7 (0.8, 9.7)	1.9 (0.6, 5.8)	0.2 (0.0, 2.6)	0.9 (0.2, 4.1)	0.7 (0.3, 1.8)

^1^ Adjusted for maternal pre-pregnancy BMI, number of years in the USA, duration of breastfeeding and birth weight.

* P-value < 0.05 based on two-sided test

BMI, body mass index; CI, confidence interval; DDE, dichlorodiphenyldichloroethylene; DDT, dichlorodiphenyltrichloroethane.

For boys, a ten-fold increase in maternal DDT and DDE concentrations was associated with a higher probability being in groups 1–3 (increasing mean growth pattern) relative to group 4 (stable mean) (Fig 1). Particularly, the estimated relative risk ratios for a ten-fold increase in serum concentration (ng/g of lipid) were greatest for group 2 (*o,p*’-DDT adj-RRR = 5.1 [95% CI: 0.5, 55.2]; *p,p*’-DDT adj-RRR = 2.9 [95% CI: 0.7, 12.4]; *p,p*’-DDE adj-RRR = 2.7 [95% CI: 0.8, 9.7]) and for group 3 (*o,p*’-DDT adj-RRR = 3.1 [95% CI: 0.3, 34.7]; *p,p*’-DDT adj-RRR = 2.1 [95% CI: 0.5, 8.8]; *p,p*’-DDE adj-RRR = 1.9 [95% CI: 0.6, 5.8]) with reference to group 4. The boys with higher levels of maternal serum concentration of DDT and DDE had a higher probability of having a stable BMI pattern in early childhood that starts to increase around 4–5 and 6–7 years of age. However, none of the adjusted associations were statistically significant (Table 4).

Similar to age-specific results reported previously at age 9 years [18], the impact of chemical exposure on developmental pattern seemed to be sex-dependent. For the girls, a ten-fold increase in prenatal DDT and DDE concentrations was generally associated with a lower probability of being in the increasing BMI growth patterns relative to the stable group 4, and some of the associations were statistically significant after adjustment (Table 4). Group 1, the linearly increasing pattern group, had the smallest estimated risk ratio relative to group 4, meaning that higher maternal DDT and DDE concentrations were associated with a lower probability of being in group 1 relative to the stable group 4. Group 3 also had a slightly lower risk relative to the stable group 4, and group 2 was similar to the reference group in terms of DDT and DDE concentrations.

In addition to maternal DDT concentrations, BMI growth pattern groups differed in terms of maternal BMI, maternal duration of residence in the U.S., breast-feeding duration, and birth weight. Children of obese mothers were most likely to be in group 1, followed by group 2, and then group 3. Lower birth weights were associated with the flat growth pattern (group 4). Shorter maternal duration of residence in the U.S. was associated with group 3 in boys and group 3 and 4 in girls. For girls in particular, a shorter duration of breast-feeding was associated with the linearly increasing pattern of group 1.

In a sensitivity analysis excluding children who were born with a low-birth weight, the results were very similar. However, when excluding children who were born preterm, the evidence from our observations was strengthened. The magnitude of the estimates was greater but the significance levels were similar.

## Discussion

A data-driven analysis of growth patterns of this birth cohort of Mexican-American mother and child pairs provides evidence that in utero exposure to DDT and DDE may impact physical development and thus obesity risk later in life, especially in boys. The heterogeneity in developmental patterns in the study population was approximated by four general mean patterns for each sex: 1) linearly increasing, 2) stable and increasing at age 4 to 5, 3) stable and increasing at age 6 to 7, and 4) flat and stable from age 2 until age 9. The trajectories start with a similar growth pattern at an early age but quickly diverge, with differences becoming greater with increased age.

Although the trajectories are data-driven rather than clinically chosen, they bear some similarities to the clinical BMI percentiles for young children [41]. For both boys and girls, group 4, which was slightly curved but largely flat overall, had a developmental pattern similar to those tracking the 50th percentile in the clinical charts. Groups 3 and 2 had growth rates that exceed that in the later years. The children in group 1 are likely to already be overweight or obese at age 2, with their BMIs continuing to increase rapidly and linearly throughout childhood.

While the mean BMI growth patterns were similar for boys and girls, the associations of these patterns with in utero DDT and DDE exposure were sex-specific. In particular, higher maternal *o,p*’-DDT and *p,p*’-DDT serum concentrations during pregnancy were significantly associated with a stable and then increasing growth pattern for boys (groups 2 and 3) and the stable pattern for girls (group 4), before adjustment. However, the magnitude of association was diluted in boys and strengthened in girls after adjusting for baseline covariates. Interestingly, prenatal DDT and DDE were not strongly associated with the linearly increasing pattern (group 1), suggesting that children in that developmental group were already on a steadily increasing BMI trajectory early in life that was largely unaffected by prenatal exposure. Rather, other genetic or behavioral factors, measured by maternal pre-pregnancy BMI and breast-feeding duration could be the driving force behind the steep, linear developmental pattern.

The existence of a sex-dependent relationship is consistent with a previous analysis based on BMI z-scores; the effect modification was not apparent at age 7 [20] but significant at age 9 [18]. This difference has been noted in 6.5–7 year olds in other studies [12, 21] that have explored effect modification of DDT by sex. However, our risk ratios are similar between the three variants of DDT and DDE within sex. This is in contrast to a previous study that suggested that the impact of sex differs for DDT and DDE [12].

The estimated risk ratios also suggest that there is a complex relationship between in utero DDT and DDE exposure and growth of BMI over time. High exposure was not associated with the greatest growth rate (group 1) but rather with stable and then moderate rates of change starting around ages 4–7, in boys. This nonlinear relationship is reminiscent of the non monotonic increase in overweight risk with highest BMI levels observed at the middle exposure levels reported by Valvi et al [12]. While we cannot directly compare results, the association we observed between exposure and the growth pattern could potentially help explain the fact that the highest exposure levels were not associated with the highest overweight risk.

A major strength of this study is fully utilizing the longitudinal nature of the BMI data and modeling the heterogeneity in developmental patterns in a data-driven manner using a finite mixture model with a data transformation. Using groups to model the variability in growth over time provides the flexibility to have non-linear relationships with baseline risk factors such as in utero exposures to *o,p*’-DDT, *o,p*’-DDT and *o,p*’-DDE. Previous analyses of these data and other longitudinal developmental studies have focused on the associations with the BMI level while accounting for the repeated measures while this study focused on identifying distinct developmental patterns over time and their relationships with exposure and other risk factors as growth patterns drive long-term levels and obesity risks [18, 20].

Other strengths of this study include the study population from the CHAMACOS study as it is relatively homogenous in terms of diet, breastfeeding, country of origin, and socioeconomic status, which can mitigate some possible sources of confounding. Otherwise, we adjusted for many measured confounders. However, there could be other baseline factors that could confound the relationship between DDT or DDE exposure and BMI development so we are careful to only interpret our results in terms of associations and not causal relationships. It should be noted that DDT and DDE concentrations measured in maternal serum likely reflect exposure many years earlier in Mexico, where DDT was used until the year 2000. Thus, the children in this study were only exposed to DDT and DDE from their mothers when they were in utero and in infancy via breastfeeding but not during childhood. However, the children continue to have measurable, albeit decreasing DDT and DDE concentrations in their blood. Thus, it is possible that changes in BMI trajectories are due to childhood DDT or DDE exposure rather than in utero exposure.

To the best of our knowledge, this is the first study to explicitly focus on the relationship between in utero exposures and growth patterns, rather than the BMI level. While we believe that BMI levels have clinical significance, the growth is a characteristic that should be explored on its own to complement studies on BMI level.

In summary, this novel analysis suggests that in utero DDT and DDE exposures may be associated with BMI increases between ages 4 and 7 among boys with previously stable BMI trajectories. Interestingly, high exposures to DDT and DDE were less associated with being in a trajectory of linearly increasing BMI beginning at age 2 or earlier, suggesting that the pattern of rapid, linear BMI gain is determined by genetic or family lifestyle factors rather than DDT or DDE exposure. We found that these endocrine disruptors exposures were associated with early childhood physical development in a non-linear manner and that sex may impact the relationship. We encourage investigators to examine growth patterns of BMI over time, in addition to BMI at specific time points to get a fuller sense of the biological mechanisms. Further longitudinal research in other populations is needed to confirm the patterns observed here.
